# Classification of accelerometer wear and non-wear events in seconds for monitoring free-living physical activity

**DOI:** 10.1136/bmjopen-2014-007447

**Published:** 2015-05-11

**Authors:** Shang-Ming Zhou, Rebecca A Hill, Kelly Morgan, Gareth Stratton, Mike B Gravenor, Gunnar Bijlsma, Sinead Brophy

**Affiliations:** 1 College of Medicine, Swansea University, Wales, UK; 2 College of Engineering, Swansea University, Wales, UK

**Keywords:** physical activity, sedentary behaviour, non-wear, accelerometer, PUBLIC HEALTH, classification

## Abstract

**Objective:**

To classify wear and non-wear time of accelerometer data for accurately quantifying physical activity in public health or population level research.

**Design:**

A bi-moving-window-based approach was used to combine acceleration and skin temperature data to identify wear and non-wear time events in triaxial accelerometer data that monitor physical activity.

**Setting:**

Local residents in Swansea, Wales, UK.

**Participants:**

50 participants aged under 16 years (n=23) and over 17 years (n=27) were recruited in two phases: phase 1: design of the wear/non-wear algorithm (n=20) and phase 2: validation of the algorithm (n=30).

**Methods:**

Participants wore a triaxial accelerometer (GeneActiv) against the skin surface on the wrist (adults) or ankle (children). Participants kept a diary to record the timings of wear and non-wear and were asked to ensure that events of wear/non-wear last for a minimum of 15 min.

**Results:**

The overall sensitivity of the proposed method was 0.94 (95% CI 0.90 to 0.98) and specificity 0.91 (95% CI 0.88 to 0.94). It performed equally well for children compared with adults, and females compared with males. Using surface skin temperature data in combination with acceleration data significantly improved the classification of wear/non-wear time when compared with methods that used acceleration data only (p<0.01).

**Conclusions:**

Using either accelerometer seismic information or temperature information alone is prone to considerable error. Combining both sources of data can give accurate estimates of non-wear periods thus giving better classification of sedentary behaviour. This method can be used in population studies of physical activity in free-living environments.

**Strengths and limitations of this study**

- This study combines movement information and skin surface temperature information to significantly improve the global performance of wear and non-wear classification in monitoring physical activity.
- This study is one of the very few studies on identifying wear time and non-wear time events using raw accelerometry data.
- This study conducted high validity in classification, which might be especially important for cohort studies that investigate the link between baseline physical activity assessment and health risks and disease outcomes over long time periods.
- This technique is only applicable to accelerometers with a temperature sensor, however, such types of accelerometers are gaining popularity.
- The temperature threshold identified in this study is UK-specific, repeated validation of optimal temperature thresholds should be undertaken in countries of different climates.

## Introduction

Increasing evidence has shown that physical activity (PA) has strong positive associations with health.^1–6^ For example, there are significant health benefits from engaging in at least 150 min of moderate-intensity activity or 75 min of vigorous-intensity activity per week.^6^ On the contrary, sedentary lifestyles and physical inactivity have proved to be the major contributors to childhood obesity, cardiovascular disease and type 2 diabetes mellitus in adults.^7^ ^8^ Accurate and reliable assessment of PA is crucial to investigating and understanding the relationship between PA and health.^9^ ^10^ The complex nature of PA in a free-living setting makes it difficult to accurately measure all of its aspects and assess the impact on outcome parameters, such as energy expenditure,^2^ outside laboratory settings.^2^ ^9–12^ A robust and comprehensive measure of PA that is applicable to surveillance, epidemiology, clinical and intervention research, still does not exist.^13^

Ideally, a valid, feasible assessment of PA should be conducted with minimal discomfort to the participant.^2^ In medical and sport science research, body-worn accelerometers are used to provide objective measurements of PA duration, frequency and intensity. However, accelerometers collect data continuously even during periods of non-wear (ie, periods when participants may not be wearing their monitor, such as during sleeping, showering and aquatic activities, and periods when participants forget to reattach a monitor). Therefore, it is challenging to distinguish times of sedentary behaviours (eg, watching television) and times of non-wear.^14–18^ Misclassification of wear time and non-wear time will result in unnecessary loss of valuable data and errors in estimates of PA level, which, in turn, could lead to errors in estimates of energy expenditure, and thus bias in understanding the relationship between PA patterns and health outcomes.

Automated estimation of accelerometer wear time and non-wear time is particularly desirable for large cohort studies, but such algorithms have not yet been standardised and their accuracy needs to be enhanced.^15^ ^16^ ^19^ A number of previous studies have sought to classify non-wear time. For example, estimation of wear time and non-wear time for dual-axis accelerometers, such as the Omron HJ-720-ITC pedometer, has been conducted by recording daily step counts.^20^ However, such a rule of thumb was only applicable to pedometers and only in terms of days, with no way of identifying a finer time resolution, such as minutes. A wide range of criteria have been suggested to automatically detect wear time and non-wear time for uniaxial accelerometers. For example, a recommendation from ActiGraph is to consider bouts of consecutive zero activity ‘counts’ for 20 min or more as non-wear, but the definition on the length of the bouts of consecutive zero activity ‘counts’ as non-wear can be arbitrary.^16^ ^18^ ^21–23^ In addition, the classification of non-zero counts can be made problematic by artefactual movements during non-wear periods; for example, a monitor left unworn in a moving car could record acceleration data similar to that of a monitor worn by a passenger in a moving car, or prolonged periods of motionless sitting may appear non-wear. Hutto *et al*^19^ have estimated wear and non-wear time for the energy expenditure and step count monitor, Actical accelerometer, in older adults. Their estimates of wear and non-wear time were based on different time lengths of consecutive zeroes, such as 60, 90, 120, 150 and 180 min, in comparison with estimates derived from log sheets. Their study indicated that using at least 120 min of consecutive zero counts can provide dependable estimates of wear and non-wear time in older adults wearing the Actical accelerometer.^19^

To our knowledge, currently, only two methods have been proposed to classify wear time and non-wear time for triaxial accelerometers.^24^ Using the wrist-worn, triaxial STMicroelectronics (GENEA) accelerometer, the first method was based on SD of the three axes of acceleration for consecutive blocks of 30 min^25^ and modified 60 min.^26^ A similar procedure was applied to the triaxial GeneActiv accelerometer for detecting non-wear time events.^27^ The second method of classifying non-wear time, proposed by Choi *et al*,^16^ ^24^ used the wrist-worn, triaxial Actigraph GT3X and was intended to improve the accuracy of a previously developed algorithm designed for the uniaxial Actigraph GT1M monitor. This method used a 90 min time window (as detection criteria for wear time and non-wear time classification) and vector magnitude counts (ie, three axes measurements), and was reported to reduce misclassification of sedentary behaviour as non-wear compared with the previous (uniaxial) algorithm. However, such estimations may misclassify non-wear periods that are shorter than the minimum length of the algorithm criteria (eg, <90 min) or wear periods (eg, external movement but no meaningful PA has occurred).^28^

In terms of types of accelerometry data, the majority of the existing automated methods for classifying wear time and non-wear time events focused on the ‘count’ accelerometry data. A count is a manufacturer-dependent output value, an arbitrary unit aimed to be proportional to the average acceleration over a selected time interval (epoch).^29^ Counts are inherently neither meaningful nor interpretable. It is often unclear what a count truly means, physically or physiologically, as the underlying data processing method and assumptions are often concealed from the end user. Moreover, the process of computing the counts often leads to loss of valuable information. In contrast, only a very few studies have been based on raw accelerometry (eg, movement data in X-Y-Z directions in g).^25^ The raw accelerometry data facilitate easier interpretation to enable the end users to gain greater control over data processing, so that more efficient methods can be developed. Therefore, there are clear demands to develop an automated classification algorithm to define wear time and non-wear time that minimises the potentials for misclassification error due to intermittent monitor removal and short periods of sedentary time.

In this paper, we develop an automated wear time and non-wear time estimation algorithm that uses objective raw acceleration data in combination with a novel measure—surface skin temperature—in order to enhance the detection of intermittent monitor removal and improve overall classification performance.

## Materials and methods

### Study participants

Fifty participants were recruited in two phases. Participants in phase 1 were used to design the algorithm (including seeking optimal parameters), and those in phase 2 to validate the algorithm. The participants wore the accelerometers in their own homes while attending to their normal daily activities, and were instructed to wear the accelerometers firmly on the skin surface even during sleep. They were asked to wear or remove accelerometers at intervals lasting at least 15 min. Participants synchronised the clocks they used with the clocks in the accelerometers before wearing them, and kept diaries of the exact timings of accelerometer wear and removal, which were compared with the predictions derived by the classification method for performance evaluation. Parents completed and maintained the diaries for those children aged under 10 years. The study had approval from the Multicentre Ethics Committee for Wales and participants gave consent (or parental assent in the case of children) to be included in the study.

### Accelerometer: GeneActiv watch

In this study, we used the triaxial accelerometer watch—*GeneActiv*, produced by ActivInsight Ltd. This monitor provides the end user with raw accelerometry data (ie, unfiltered acceleration signals) in three axes (forward/backward, up/down, left/right), therefore offering the end user greater control over data processing, and a measure of surface skin temperature. The *GeneActiv* was chosen because it is portable, non-intrusive and waterproof, and therefore suitable for continuous 24 h use in free-living conditions. The size of the *GeneActiv* is 43 mm×40 mm×13 mm, and the weight is 16 g (without strap). The unit can be worn on the ankle by young children to ensure it remains in place and is not easily or unwittingly removed, or by working adults when employment regulations restrict the wearing of watches. These properties make it particularly suitable for monitoring both child and adult PA. Its reliability and validity have been extensively assessed.^27^ ^30^ The *GeneActiv* contains sufficient memory to store up to 60 days of data when sampling at 10 Hz frequency, or 7 days at 100 Hz. When activated, the *GeneActiv* continuously tracks and stores activity information whether or not it is worn. An example of its output is illustrated in online supplementary table S1.

For the purposes of this study, the accelerometer was worn on the wrist by adults and, in order that it not be easy to remove, on the ankle by children. The requirement for wearing or removing accelerometers at intervals lasting at least 15 min by participants is to make sure the accelerometer temperature measurements correctly reflect the changes of events—wear and non-wear, respectively.

All accelerometers had resin straps. Acceleration was sampled at 100 Hz (maximum sampling frequency) and data were stored in gravity units for offline analyses. There are no missing data in the data sets collected from the two phases.

### Time series analysis and algorithm development

#### Temperature only

Our pilot data showed that patterns of accelerometer temperature vary systematically along with wear and non-wear status (see online supplementary figure S1). In most cases, temperatures increased during wear, and decreased after the accelerometer was removed. This was, therefore, the basis for our classification algorithm.

Three steps are required to examine the lingering effects of preceding temperature values. The first step is to use the autocorrelation function (ACF) to examine the patterns of lingering effects of preceding temperatures. The ACF measured the internal association between temperature observations at different times (see online supplementary figure S2). For example, choosing an arbitrary spot in PA time, one might ask: “On the average, what does the temperature time series look like in 30 min time of PA, compared to now?” This is a question about how strong the internal association within the series is at a period of 30 min. Such an association could be very strong and positive (ie, the series in 30 min is similar to now), or be very strong and negative (ie, the series in 30 min is very dissimilar to now) or there could be a weak or no relationship (ie, there is no identifiable association). ACF provides an important guide to the persistence in an accelerometer temperature time series. It quantifies the degree of internal association between observations at different times using a value within the interval (−1 to 1): a value of +1 indicates a strong positive association, a tendency for a system to remain in the same state from the past values of one observation to now; −1 indicates a strong negative association, a tendency for a system to evolve in the different state at one observation from the past values; and 0 indicates no association between one observation and its past values. The distinct cut-off of the ACF of the temperature time series suggests that a moving average model might be appropriate for these data, particularly, the accelerometer temperature was heavily subjected to noise or rapid fluctuations caused by routine activities (eg, cooking with hot implements, or washing hands with cold or hot water). In the second step, the low-pass filter (moving average model) was used to smooth the data and remove short-term temperature fluctuations. The *moving* average was determined using the mean of the previous historical temperature sample (K)*.* It can be considered as a moving window technique, with output obtained by multiplying the signal by a rectangular window, the height of which is 1/K, which moves as the time step is updated (see online supplementary figure S3). In the third step, temperature data were then used to construct a classifier for detecting the trends. Let T_t_ represent the smoothed accelerometer temperature at time t. First, a threshold temperature T_0_ was defined, so that above the T_0_, the accelerometer was classified as wear. For a temperature below the T_0_, the classification was carried out based on the trend of temperature changes. In general, if there was an increasing trend, the accelerometer was classified as wear; if the trend was decreasing, the classification was non-wear. The key and difficult task was to identify the trends of the temperature changes that are not masked by short-term fluctuations.

**Figure 1:**
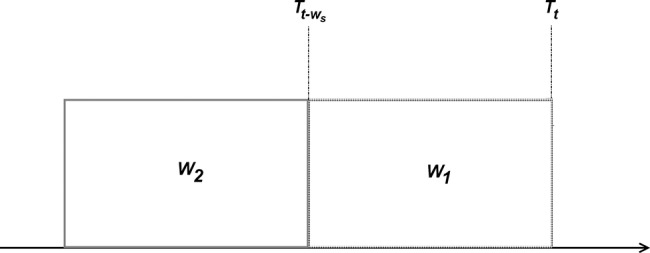
*Bi-moving-window for classifying trends in temperature change*: W_1_ represents the window at time t, and the time period over which the observed temperature at t is smoothed (moving window). W_2_ represents the window at time t−w_s_. A difference between (smoothed) temperatures calculated in each window is used to indicate an increasing or decreasing temperature trend (avoiding spurious conclusions due to very short-term fluctuations). Both W_1_ and W_2_ are of equal size w_s_.

Our detections of ‘increasing’ or ‘decreasing’ trends were based on two moving average windows (bi-windows) of temperature (see figure 1). To assess whether the temperature at time t (T_t_) followed an increasing or decreasing trend, the temperature T_t_ was compared with the previous temperature 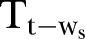
at time t−w_s_, where w_s_ represented the size of window W_1_. Note that the value of T_t_ was the *average* of the temperatures within the window W_1_, and the value of 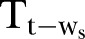 the *average* of the temperatures within the window W_2_. The size of window W_2_ was the same as the W_1_. Then, classification rules based on temperature alone are as depicted below.

1. If 
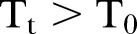
 the accelerometer is classified as ‘wear’.
2. If 
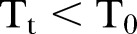
 and if 
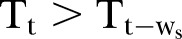
, the trend of the temperature change is assessed as increasing, and the accelerometer is classified as ‘wear’.
3. If 
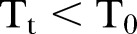
 and if 
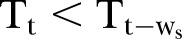
, the trend of the temperature change is assessed as decreasing and the accelerometer is classified as ‘non-wear’.
4. If 
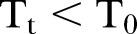
 and 
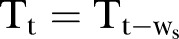
, the status of the accelerometer remains unchanged.

#### Acceleration only

With the seismic acceleration alone in the three axes, the wear time and non-wear time was detected in previous studies.^25^ ^27^ A block of accelerometer was classified as a non-wear if the SD of acceleration values in the current moving window was less than 13 mg (1 mg=0.00981 m/s^2^) and the value range less than 50 mg for at least two out of three axes. These thresholds were chosen according to acceleration signals for a large cohort study using *GeneActiv* accelerometers.^27^

#### Combining temperature and acceleration

We combined accelerometer temperature and acceleration data (CTA) to develop the automated wear time and non-wear time detection algorithm as follows:

1. An event was classified as non-wear if the temperature T_t_ was below the T_0_ and the SD of seismic acceleration values in the current moving window W_1_ was less than 13 mg for the three axes.
2. An event was classified as wear if the temperature T_t_ was above the T_0_. The algorithm under this case is very useful in some scenarios, such as watching TV, sleeping, etc, where movement would be ‘low’ while accelerometer temperature could be ‘high’.
3. For cases where the temperature was below T_0_, but the SD of seismic acceleration values was greater than 13 mg, the classification of wear or non-wear was made in terms of the smoothed temperature trends (as described above in case 1).

**Figure 2:**
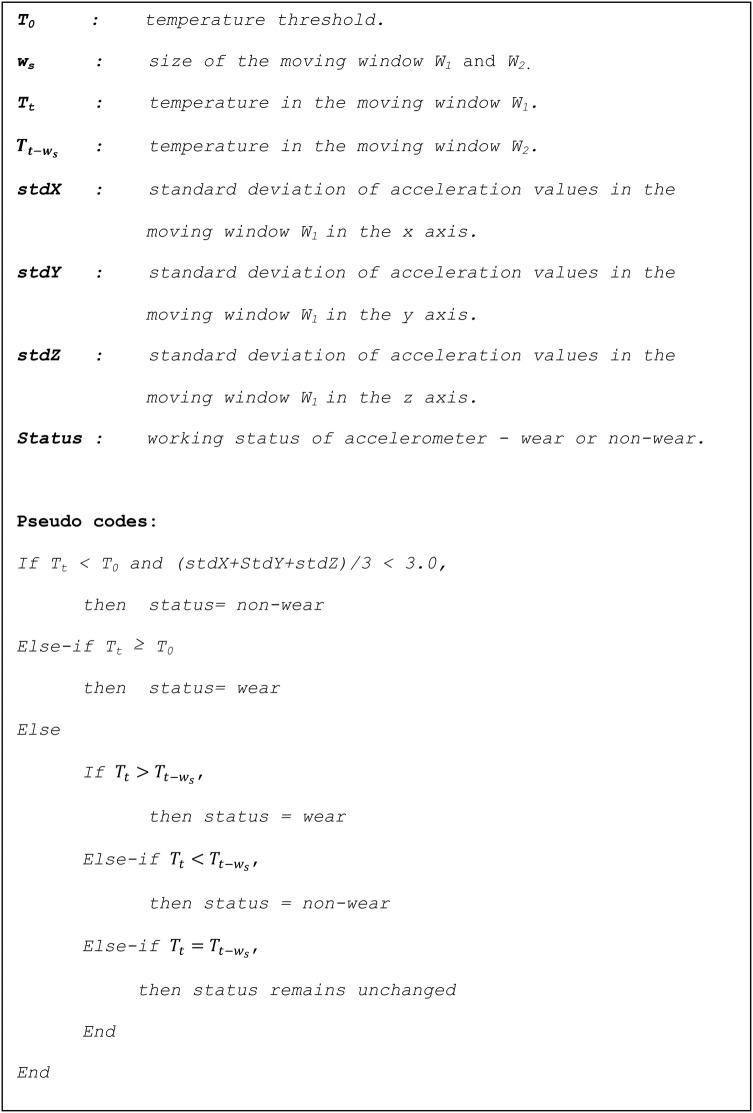
*Pseudo codes for proposed algorithm to* classify wear and non-wear events based on acceleration, temperature and trends in temperature.

Figure 2 depicts the pseudo codes for implementing the CTA method.

In this study, we set w_s_ (the size of the window) at 1 min for the algorithms of temperature only, acceleration only and CTA described above, and moving window is updated forward at every time step by 1 s. It is noted that the size of moving window in this study is different from the ones in previous studies.^25–27^ In addition, although it is required to wear or remove accelerometers at intervals that last at least 15 min, these first 15 min data about each wear or non-wear event are included in the study.

### Parameter estimation and validation of the proposed method

The algorithms relied on certain parameters for which values were provided by the user, and the classifier's performance was compared with known periods of wear and non-wear from the time-log diaries. These two exercises were conducted on separate data sets in this study. In each of the following investigations, our data set from phase 1 was used to design the algorithm (to estimate the optimal parameters), and data set from phase 2 was used as an independent assessment of the performance of the proposed method.

The classifier's performance was assessed in terms of *sensitivity* (the proportion of actual wear events correctly identified by our model), *specificity* (the proportion of actual non-wear events correctly identified by our model), *positive predictive value* (PPV) (the proportion of predicted wear events that were truly wear), *negative predictive value* (NPV; the proportion of predicted non-wear events that were truly non-wear) and classification rate (CR), where the CR was calculated as the ratio of true positive rate (TPR) and true negative rate to the total sampling points.

In considering the acceleration threshold parameter to be fixed at the standard value (from previous studies^25^), two parameters needed to be defined for our algorithm: the size of the moving window w_s_, and the temperature threshold T_0_. Since our aim was to develop a classifier that can be applied in real time with minimal restrictions, we fixed a priori the value of w_s_ (size of the window) at 1 min (ie, using 1 min of PA data points to predict the current accelerometer status). This left only the T_0_ to be estimated from the phase 1 data.

**Figure 3:**
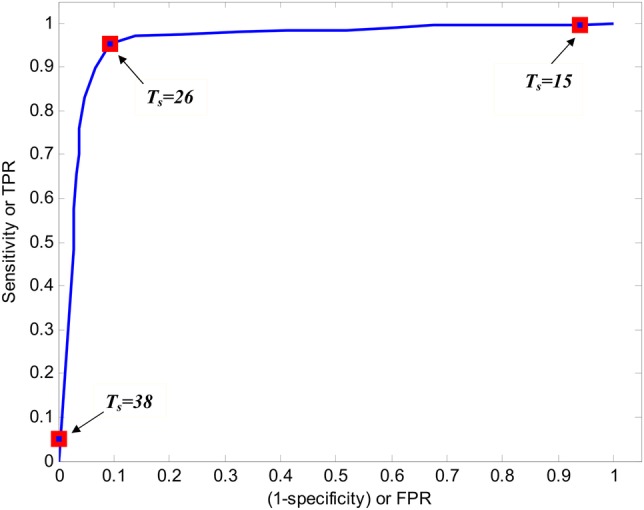
Receiver operating characteristic curve for seeking optimal temperature threshold (FPR, false positive rate; TPR, true positive rate).

Clearly, the classification performance depended on T_0_, with an important trade-off between sensitivity and specificity. In general, it is intuitive that using a high temperature threshold will lead to good specificity (non-wear is unlikely to be misclassified as wear) but poor sensitivity (wear will often be incorrectly classified as non-wear). In contrast, using a low temperature threshold will lead to high sensitivity (wear is unlikely to be misclassified) but at the expense of low specificity (non-wear will often be misclassified). To strike a balance, a receiver operating characteristic (ROC) plot was constructed for the relationship between *sensitivity* and *1-specificity*, generated by applying the algorithm with a range of T_0_ values (15–38°C) to the pooled wear/non-wear events from the training phase 1 data set. The top left corner of the ROC plot represents the perfect classification with 100% sensitivity (no false negatives) and 100% specificity (no false positives). Therefore, the best value of T_0_ was identified as that point on the ROC curve nearest the top left coordinate (ie, high sensitivity without losing too much specificity)^31^ (see figure 3), which would give the highest sum of sensitivity and specificity.

After identification of the parameter T_0_, the *Temperature* alone based method in (1) and CTA method in (3) with the optimal T_0_ were applied to the validation samples (phase 2, n=30) separately. The algorithm based on only acceleration in (2) does not need more hyperparameters to be identified, except the known threshold. The sensitivity, specificity, and PPV and NPV values were calculated for all events on an individual participant level, and these values were averaged (with 95% CIs) to form our overall assessment of model performance. Comparisons of performance were made between subgroups of children and adults, and between males and females (with 0.01 significance level), using Behrens-Fisher statistical test^3^ ^32^ (for which no assumptions of equal variances were made).

## Results

### Data collection

The 50 participants, 30 females and 20 males, included 23 children (aged under 16 years) and 27 adults (aged 17 years and older). The 20 participants (including 7 children) were recruited in phase 1 and the rest recruited in phase 2. Our algorithm aims to classify the wear time and non-wear time events in seconds. Once an accelerometer was switched on, the data collection started to record its working condition, no matter whether it was worn by a participant or not. The total 728.6 h of working conditions of the accelerometers allowed us to collect 2 622 974 accelerometer data samples in seconds for this study, among which the wearing periods had 716 983 samples (199.2 h) and non-wear periods 1 905 991 samples (529.4 h).

### Temperature autocorrelation

The ACF characterises the persistence of the state of observations within time series. The accelerometer temperatures demonstrate the strong ‘increasing’ or ‘decreasing’ trends along with the variations of the wear time and non-wear time status. The left plot of online supplementary figure S2 depicts an example of time series of the accelerometer temperatures with over 15 h, while the right plot shows the corresponding empirical autocorrelations of this time series (the correlogram), in which the dashed lines are the 95% confidence bands. The autocorrelation at lag zero is always one. This is because a series is always perfectly correlated with itself. At lag 1–128, the autocorrelation values are very close to 1, which means that temperatures given at 2 min intervals are very similar to each other (either before or after). Indeed, all autocorrelation up to lag 982 (seconds) are greater than 0.8, indicating highly linear association up to 16.4 min apart. At the 95% confidence level, the autocorrelations at lag 1 up to 111.9 min demonstrate statistically significant positive associations. A different picture emerges when looking at instances of time separated by a larger number of lags. The autocorrelation taken 115 min apart is −0.013. This indicates that the observations of temperature 115 min apart are dissimilar.

### Algorithm temperature threshold

Inspection of the ROC curve for the algorithm's performance (on phase 1 data) resulted in an optimal estimate of T_0_=26°C, achieving the performance of sensitivity 0.954, specificity 0.906 and overall CR 0.941 (see figure 3).

### Validation performance: sensitivity and specificity

Averaging the classification performances over all participants in the validation set, table 1 summarises them in terms of CR, sensitivity, specificity, PPV and NPV. The proposed CTA classifier achieved an overall sensitivity of 0.94 (95% CI 0.90 to 0.98), specificity of 91% (95% CI 0.88% to 0.94%), PPV as 0.81 (95% CI 0.75 to 0.88) and NPV as 0.95 (95% CI 0.89 to 1.00). In terms of average misclassifications per day, the proposed CTA method achieved 53.1 misclassified minutes per day, while the acceleration alone method generated 257 misclassified minutes per day and the temperature alone method produced 468 misclassified minutes per day. For all performance characteristics, the statistical t test showed that the proposed CTA method achieved considerable improvements on the classification performance over the existing methods that use acceleration alone (p=0.009) or temperature alone (p<0.001).

**Table 1:** The performance of classifying wear and non-wear events: mean and 95% CIs

Method	CR (95% CI)	Sensitivity (95% CI)	Specificity (95% CI)	PPV (95% CI)	NPV (95% CI)
The proposed CTA method	0.95 (0.92 to 0.97)	0.94 (0.90 to 0.98)	0.91 (0.88 to 0.95)	0.82 (0.76 to 0.88)	0.95 (0.89 to 1.00)
The temperature alone	0.75 (0.67 to 0.82)	0.93 (0.90 to 0.98)	0.64 (0.59 to 0.69)	0.53 (0.36 to 0.69)	0. 95 (0.89 to 1.00)
The acceleration alone	0.80 (0.71 to 0.90)	0.76 (0.64 to 0.89)	0.89 (0.84 to 0.94)	0. 75 (0.67 to 0.83)	0.74 (0.60 to 0.89)

CR, classification rate; CTA, combining temperature and acceleration; NPV, negative predictive value; PPV, positive predictive value.

#### Children versus adults

Table 2 summarises the classification performances for children versus adults. In children, the proposed CTA method led to a sensitivity of 0.96 (95% CI 0.94 to 0.99) and specificity of 0.93 (95% CI 0.90 to 0.96). As a comparison, the acceleration alone classifier^25^ achieved sensitivity 0.76 (95% CI 0.6 to 0.93) and specificity 0.89 (95% CI 0.84 to 0.94), the temperature alone classifier generated sensitivity 0.96 (95% CI 0.93 to 0.99) and specificity 0.64 (95% CI 0.59 to 0.69). In adults, the CTA method sensitivity was 0.93 (95% CI 0.88 to 0.98) and specificity 0.88 (95% CI 0.82 to 0.94). Thus, the CTA method performed equally well for the groups of children and adults. No statistically significant difference was found between classification of children's events (wear time and non-wear time) and adults’ events by the CTA method (p=0.36).

**Table 2:** The performance of classifying wear and non-wear events for children versus adults

Method	CR (95% CI)	Sensitivity (95% CI)	Specificity (95% CI)	PPV (95% CI)	NPV (95% CI)
Children (5–16 years)					
The proposed CTA method	0.96 (0.95 to 0.98)	0.96 (0.93 to 0.99)	0.94 (0.91 to 0.97)	0.79 (0.70 to 0.89)	0. 97 (0.93 to 1.00)
The temperature alone	0.73 (0.64 to 0.82)	0.96 (0.93 to 0.99)	0.64 (0.59 to 0.69)	0.45 (0.24 to 0.67)	0.97 (0.93 to 1.00)
The acceleration alone	0.79 (0.65 to 0.92)	0.73 (0.55 to 0.92)	0.89 (0.85 to 0.94)	0.69 (0.60 to 0.79)	0.75 (0.55 to 0.95)
Adults (≥17 years)					
The proposed CTA method	0.93 (0.88 to 0.98)	0.90 (0.82 to 0.99)	0.88 (0.82 to 0.94)	0.86 (0.78 to 0.93)	0.92 (0.78 to 1.00)
The temperature alone	0.77 (0.64 to 0.9)	0.90 (0.82 to 0.99)	0.65 (0.55 to 0.75)	0.63 (0.37 to 0.9)	0.92 (0.79 to 1.00)
The acceleration alone	0.83 (0.70 to 0.96)	0.80 (0.65 to 0.97)	0.89 (0.79 to 0.99)	0.83 (0.70 to 0.95)	0.74 (0.51 to 0. 97)

CR, classification rate; CTA, combining temperature and acceleration; NPV, negative predictive value; PPV, positive predictive value.

#### Females versus males

Table 3 summarises the classification performances for females versus males. For females, the CTA method led to a sensitivity of 0.92 (95% CI 0.86 to 0.99) and specificity of 0.91 (95% CI 0.86 to 0.96). As a comparison, the acceleration alone classifier achieved sensitivity 0.88 (95% CI 0.73 to 1.00) and specificity 0.88 (95% CI 0.78 to 0.97), the temperature alone classifier generated a sensitivity 0.92 (95% CI 0.85 to 0.99) and specificity of 0.62 (95% CI 0.54 to 0.70). For males, sensitivity was 0.95 (95% CI 0.91 to 1.00) and specificity 0.91 (95% CI 0.87 to 0.95). No statistically significant difference was found between classification of females’ events (wear time and non-wear time) and males’ events by the CTA method (p=0.75).

**Table 3:** The performance of classifying wear and non-wear events for females versus males

Method	CR (95% CI)	Sensitivity (95% CI)	Specificity (95% CI)	PPV (95% CI)	NPV (95% CI)
Females					
The proposed CTA method	0.95 (0.92 to 0.98)	0.92 (0.86 to 0.99)	0.91 (0.86 to 0.96)	0.80 (0.70 to 0.89)	0.96 (0.91 to 1.00)
The temperature alone	0.76 (0.61 to 0.84)	0.92 (0.85 to 0.99)	0.62 (0.54 to 0.70)	0.49 (0.23 to 0.75)	0.96 (0.91 to 1.00)
The acceleration alone	0.85 (0.72 to 0.99)	0.87 (0.70 to 1.00)	0.89 (0.80 to 0.98)	0.75 (0.62 to 0.88)	0.82 (0.59 to 1.00)
Males					
The proposed CTA method	0.94 (0.90 to 0.98)	0.95 (0.90 to 1.00)	0.91 (0.87 to 0.96)	0.84 (0.75 to 0.92)	0.94 (0.84 to 1.00)
The temperature alone	0.76 (0.67 to 0.86)	0.95 (0.91 to 1.00)	0.66 (0.60 to 0.72)	0.55 (0.33 to 0.78)	0.94 (0.84 to 1.00)
The acceleration alone	0.76 (0.63 to 0.89)	0.68 (0.50 to 0.85)	0.89 (0.84 to 0.95)	0.74 (0.64 to 0.85)	0.69 (0.49 to 0.89)

CR, classification rate; CTA, combining temperature and acceleration; NPV, negative predictive value; PPV, positive predictive value.

It is noteworthy that, no doubt, the children have different patterns of PA from those of adults,^33^ and females’ patterns of PA could be different from males’ patterns as well,^34^ but the events of wear time and non-wear time variations were not found to be significantly different between these corresponding groups in our study.

## Discussion

The clinical consequence of misclassification of accelerometer wear time and non-wear time would be overestimation or underestimation of the intensity level of PA and energy expenditure, thus misleading the interpretation of the relationship between PA and health outcomes.^16^ This study provides a simple and efficient methodology on use of short time periods of consecutive data blocks (1 min) to accurately predict triaxial accelerometer wear time and non-wear time status. We have rigorously assessed the method, which shows that the traditional acceleration alone method tended to predict non-wear events better than wear events, while temperature data alone predicted wear but not non-wear well. Our proposed CTA method generated an average sensitivity of 95% and specificity of 91%, and hence performed very well for both categories—significantly better than the methods of acceleration (p=0.009) and temperature (p<0.001) alone.

Using time series analysis would provide a novel way of employing multiple sensors (those of acceleration and temperature), which can improve the estimation of the accelerometer status. By generating more accurate time spent in sedentary and active behaviours in free-living conditions, this method will benefit population studies of PA to gain correct insights into PA and its impact on health, particularly paediatric population studies to capture sporadic, short bursts of PA. On the other hand, sleep is the most challenging type of activity for classification.^35^ Out pilot data showed that the temperature signals of accelerometers during participants’ sleeping remained very high, which provides a unique indication to help reduce the risk of incorrect non-wear time detection.

Our proposed CTA method has several advantages: (1) This study is one of the very few studies on identifying wear time and non-wear time events using raw accelerometry data, which are fundamentally different from the majority of studies of this kind based on count output accelerometry values. (2) The study has high validity in classification. This characteristic might be especially important for cohort studies that investigate the link between baseline PA assessment and health risks and disease outcomes over long time periods.^16^ ^36–39^ (3) The requirement for only 1 min periods of data, in contrast to existing methods based on much longer data blocks (often in the order of 30 min or more), would prove useful for research on paediatric populations to capture sporadic, short bursts of activity expected from younger participants,^11^ ^33^ in which the whole activity often does not last very long. However, without the aid of temperature signals, the use of a 1 min window for acceleration data alone would increase the risk of incorrect non-wear time detection, particularly during sleep. Our proposed CTA method offers a novel way of combining temperature and acceleration information to improve the classification of wear time and non-wear time events. (4) Reduced misclassification due to artefactual monitor movements during non-wear. (5) The potential to develop further non-wear algorithms for other accelerometers that measure skin surface temperature data (such as SenseWear Armband^40^), based on the principles of the method reported herein. (6) The potential to develop further non-wear algorithms by combining the temperature only algorithm with other acceleration only algorithms. Our current CTA simply combines the temperature only algorithm with the acceleration only algorithm used in certain previous studies.^25^ ^27^ In other words, given an acceleration only algorithm, increased accuracy of estimating wear and non-wear events is expected to gain by integrating with the temperature only algorithm described in this paper.

Our proposed method has some potential limitations for practical applications. First, this technique is only applicable to accelerometers with a temperature sensor; however, such types of accelerometers are gaining popularity. Nevertheless, this study may stimulate other manufacturers to include a skin temperature sensor standard in their accelerometers. Second, if an accelerometer is not worn firmly on the surface of the skin, for example, if it is placed over or within clothing rather than directly on the skin, the changes of temperatures may not be as useful for classification. Third, the proposed method is based on requests that participants should wear or not wear accelerometers for at least 15 min to capture adequate movement and temperature information for the method before starting classification. It is possible that performance of the classifier will not be as impressive for shorter periods, though we suggest that errors in events lasting less than this time would have a relatively small impact on conclusions drawn from population level studies. Fourth, despite concerns for participant burden and the potential for classification error, the use of time-log diaries to record periods of wear time and non-wear time was deemed appropriate within this study due to its relatively short duration. Longer term validation studies would likely benefit from using direct observation by researcher or video as the ‘gold standard’ method of observing PA. Finally, for validation of the temperature threshold (eg, UK-specific 26°C), one needs to notice the impacts of some temperature variations on classification, such as in indoor activities and outdoor activities by the same person, situations where temperature changes across seasons. One possible solution is to perform multiple-fold cross-validations for the best compromised threshold to aid the acceleration information for improving classification. Therefore, repeated validation of optimal temperature thresholds should be undertaken in different situations and countries with different climates. Nevertheless, the benefits offered by combining temperatures with acceleration information would exceed them, in particular, the increased accuracy to be gained. It is noted that when more information is introduced, advanced system modelling techniques^41–43^ may be needed to tackle increased tasks, such as model overfitting, model transparency, etc, in classifying the types of PA.

## Conclusion

Using data either on acceleration or skin temperature alone is inadequate to correctly classify periods of wear and non-wear in some scenarios under a free-living condition. Combining both types of data within a simple and efficient algorithm requiring short time periods of data capture can significantly improve the wear time and non-wear time classification, and generate high accuracy in adults and children of both genders, although more work is need to modify the method of integrating acceleration and skin temperature information. More accurate estimations of time spent in sedentary and active behaviours in free-living conditions are expected by using the proposed algorithm.

## References

[1] Research WCRFAIfC. Food, nutrition, physical activity, and the prevention of cancer: a global perspective. Washington DC: American Institute for Cancer Research, 2007:198–209.

[2] Plasqui G, Westerterp KR Physical activity assessment with accelerometers: an evaluation against doubly labeled water. Obesity (Silver Spring) 2007;15:2371–9. 10.1038/oby.2007.281 17925461

[3] Fang J, Wylie-Rosett J, Cohen HW Exercise, body mass index, caloric intake, and cardiovascular mortality. Am J Prev Med 2003;25:283–9. 10.1016/S0749-3797(03)00207-1 14580628

[4] Gotay CC Behavior and cancer prevention. J Clin Oncol 2005;23:301–10. 10.1200/JCO.2005.06.069 15637393

[5] WHO. Global strategy in diet, physical activity and health. http://www.who.int/dietphysicalactivity/factsheet_inactivity/en/endex.html (accessed Jan 2013).

[6] Kern M, Reynolds C, Friedman H Predictors of physical activity patterns across adulthood: a growth curve analysis. Pers Soc Psychol Bull 2010;36:1058–72. 10.1177/0146167210374834 20573949

[7] Kriska AM, Saremi A, Hanson RL Physical activity, obesity, and the incidence of type 2 diabetes in a high-risk population. Am J Epidemiol 2003;158:669–75. 10.1093/aje/kwg191 14507603

[8] Plotnikoff RC Physical activity in the management of diabetes: population based perspectives and strategies. Can J Diab 2006;30:52–62. 10.1016/S1499-2671(06)01009-4

[9] Plasqui G, Joosen AM, Kester AD Measuring free-living energy expenditure and physical activity with triaxial accelerometry. Obes Res 2005;13:1363–9. 10.1038/oby.2005.165 16129718

[10] Troiano RP A timely meeting: objective measurement of physical activity. Med Sci Sports Exerc 2005;37 (11 Suppl):S487–9. 10.1249/01.mss.0000185473.32846.c3 16294111

[11] Abbott RA, Davies PS Habitual physical activity and physical activity intensity: their relation to body composition in 5.0-10.5-y-old children. Eur J Clin Nutr 2004;58:285–91. 10.1038/sj.ejcn.1601780 14749749

[12] Crouter SE, Clowers KG, Bassett DR Jr A novel method for using accelerometer data to predict energy expenditure. J Appl Physiol 2006;100:1324–31. 10.1152/japplphysiol.00818.2005 16322367

[13] Troiano RP Large-scale applications of accelerometers: new frontiers and new questions. Med Sci Sports Exerc 2007;39:1501 10.1097/mss.0b013e318150d42e 17805080

[14] Herrmann SD, Barreira TV, Kang M Impact of accelerometer wear time on physical activity data: a NHANES semisimulation data approach. Br J Sports Med 2014;48:278–82. 10.1136/bjsports-2012-091410. 22936409

[15] Winkler EA, Gardiner PA, Clark BK Identifying sedentary time using automated estimates of accelerometer wear time. Br J Sports Med 2012;46:436–42. 10.1136/bjsm.2010.079699 21504965 PMC3534985

[16] Choi L, Liu Z, Matthews CE Validation of accelerometer wear and nonwear time classification algorithm. Med Sci Sports Exerc 2011;43:357–64. 10.1249/MSS.0b013e3181ed61a3 20581716 PMC3184184

[17] Matthews CE, Hagstromer M, Pober DM Best practices for using physical activity monitors in population-based research. Med Sci Sports Exerc 2012;44 (1 Suppl 1):S68–76. 10.1249/MSS.0b013e3182399e5b 22157777 PMC3543867

[18] Oliver M, Badland HM, Schofield GM Identification of accelerometer nonwear time and sedentary behavior. Res Q Exerc Sport 2011;82:779–83. 10.1080/02701367.2011.10599814 22276419

[19] Hutto B, Howard VJ, Blair SN Identifying accelerometer nonwear and wear time in older adults. Int J Behav Nutr Phys Act 2013;10:120 10.1186/1479-5868-10-120 24156309 PMC4015851

[20] Richardson CR, Buis LR, Janney AW An online community improves adherence in an internet-mediated walking program. Part 1: results of a randomized controlled trial. J Med Internet Res 2010;12:e71 10.2196/jmir.1338 21169160 PMC3056526

[21] Hagstromer M, Oja P, Sjostrom M Physical activity and inactivity in an adult population assessed by accelerometry. Med Sci Sports Exerc 2007;39:1502–8. 10.1249/mss.0b013e3180a76de5 17805081

[22] Mattocks C, Leary S, Ness A Intraindividual variation of objectively measured physical activity in children. Med Sci Sports Exerc 2007;39:622–9. 10.1249/mss.0b013e318030631b 17414799

[23] Treuth MS, Sherwood NE, Butte NF Validity and reliability of activity measures in African-American girls for GEMS. Med Sci Sports Exerc 2003;35:532–9. 10.1249/01.MSS.0000053702.03884.3F 12618587

[24] Choi L, Ward SC, Schnelle JF Assessment of wear/nonwear time classification algorithms for triaxial accelerometer. Med Sci Sports Exerc 2012;44:2009–16. 10.1249/MSS.0b013e318258cb36 22525772 PMC3443532

[25] van Hees VT, Renstrom F, Wright A Estimation of daily energy expenditure in pregnant and non-pregnant women using a wrist-worn tri-axial accelerometer. PLoS ONE 2011;6:e22922 10.1371/journal.pone.0022922 21829556 PMC3146494

[26] van Hees VT, Gorzelniak L, Dean Leon EC Separating movement and gravity components in an acceleration signal and implications for the assessment of human daily physical activity. PLoS ONE 2013;8:e61691 10.1371/journal.pone.0061691 23626718 PMC3634007

[27] da Silva IC, van Hees VT, Ramires VV Physical activity levels in three Brazilian birth cohorts as assessed with raw triaxial wrist accelerometry. Int J Epidemiol 2014;43:1959–68. 10.1093/ije/dyu203 25361583 PMC4276065

[28] van Domelen DR, Koster A, Harris TB Accelerometer nonwear algorithms: optimizing parameters for both wear states. Med Sci Sports Exerc 2011;43:932 10.1249/MSS.0b013e318212b002 21499057

[29] Troiano RP Translating accelerometer counts into energy expenditure: advancing the quest. J Appl Physiol 2006;100:1107–8. 10.1152/japplphysiol.01577.2005 16540708

[30] Morgan KL, Rahman MA, Hill RA Physical activity and excess weight in pregnancy have independent and unique effects on delivery and perinatal outcomes. PLoS ONE 2014;9:e94532 10.1371/journal.pone.0094532 24722411 PMC3983184

[31] Fawcett. An introduction to ROC analysis. Pattern Recognit Lett 2006;27:861–74. 10.1016/j.patrec.2005.10.010

[32] Cochran. Approximate significance levels of Behrens-Fisher test. Biometrics 1964;20:191–5. 10.2307/2527627

[33] de Vries SI, Engels M, Garre FG Identification of children's activity type with accelerometer-based neural networks. Med Sci Sports Exerc 2011;43:1994–9. 10.1249/MSS.0b013e318219d939 21448085

[34] Barnekow-Bergkvist M, Hedberg G, Janlert U Physical activity pattern in men and women at the ages of 16 and 34 and development of physical activity from adolescence to adulthood. Scand J Med Sci Sports 1996;6:359–70. 10.1111/j.1600-0838.1996.tb00108.x 9046548

[35] Ekstedt M, Nyberg G, Ingre M Sleep, physical activity and BMI in six to ten-year-old children measured by accelerometry: a cross-sectional study. Int J Behav Nutr Phys Act 2013;10:82 10.1186/1479-5868-10-82 23800204 PMC3691618

[36] Colditz GA, Feskanich D, Chen WY Physical activity and risk of breast cancer in premenopausal women. Br J Cancer 2003;89:847–51. 10.1038/sj.bjc.6601175 12942116 PMC2394493

[37] Harrington M, Gibson S, Cottrell RC A review and meta-analysis of the effect of weight loss on all-cause mortality risk. Nutr Res Rev 2009;22:93–108. 10.1017/S0954422409990035 19555520

[38] Hu FB, Manson JE, Stampfer MJ Diet, lifestyle, and the risk of type 2 diabetes mellitus in women. N Engl J Med 2001;345:790–7. 10.1056/NEJMoa010492 11556298

[39] Rockhill B, Willett WC, Manson JE Physical activity and mortality: a prospective study among women. Am J Public Health 2001;91:578–83. 10.2105/AJPH.91.4.578 11291369 PMC1446638

[40] Body. Media Sense Wear System. http://sensewear.bodymedia.com/ (accessed Jan 2013).

[41] Zhou SM, Gan JQ Low-level interpretability and high-level interpretability: a unified view of data-driven interpretable fuzzy system modelling. Fuzzy Sets Syst 2008;159:3091–131. 10.1016/j.fss.2008.05.016

[42] Zhou SM, Lyons RA, Bodger OG Local modelling techniques for assessing micro-level impacts of risk factors in complex data: understanding health and socioeconomic inequalities in childhood educational attainments. PLoS ONE 2014;9:e113592 10.1371/journal.pone.0113592 25409038 PMC4237439

[43] Zhou SM, Gan JQ Constructing L2-SVM-based fuzzy classifiers in high-dimensional space with automatic model selection and fuzzy rule ranking. IEEE Trans Fuzzy Syst 2007;15:398–409. 10.1109/TFUZZ.2006.882464

